# Supplementary material for: Uncovering and tailoring hidden Rashba spin–orbit splitting in centrosymmetric crystals
Source: Nat Commun. 2019 Feb 22;10:906. doi: 10.1038/s41467-019-08836-4 (PMC6385307; doi:10.1038/s41467-019-08836-4)
Supplement: Supplementary file 1 — Supplementary Information [file 41467_2019_8836_MOESM1_ESM.pdf]

Supplementary Information

Uncovering and tailoring hidden Rashba spin-orbit splitting in centrosymmetric crystals

Linding Yuan[1,2†], Qihang Liu[3,4†], Xiuwen Zhang[5], Jun-Wei Luo[1,2,6]*, Shu-Shen Li[1,2,6], and Alex Zunger[3]*

[1] *State Key Laboratory for Superlattices and Microstructures, Institute of Semiconductors, Chinese Academy of Sciences, Beijing 100083, China*

[2] *Center of Materials Science and Optoelectronics Engineering, University of Chinese Academy of Sciences, Beijing 100049, China*

[3] *Renewable and Sustainable Energy Institute, University of Colorado, Boulder, CO 80309, USA*

[4] *Institute for Quantum Science and Engineering and Department of Physics, Southern University of Science and Technology, Shenzhen 518055, China*

[5] *College of Electronic Science and Technology, Shenzhen University, Guangdong 518060, China*

[6] *Beijing Academy of Quantum Information Sciences, Beijing 100193, China*

Supplementary Information includes:

Supplementary Notes 1-3

Supplementary Figure 1

Supplementary Table 1

Supplementary Note 1: Identification of the two inversion-partner sectors in ferroelectric SnTe and illustration of how the R-1 spin-splitting originates from the local symmetries of the sectors rather than being a consequence of the global inversion symmetry breaking.

At room temperature, α-SnTe has the centrosymmetric rocksalt (space group Fm-3m) structure[1] (Supplementary Figure 1a) and, as temperature is lowered,[2] it undergoes a (ferroelectric) phase transition to the non-centrosymmetric rhombohedral (space group R3m) R-1 structure,[3,4] where the Te atom is spontaneously displaced along the [111] direction relative to Sn atom. We focus on the bands near Fermi energy around the Z point which exhibits the most pronounced Rashba spin splitting (Supplementary Figure 1b).

In the centrosymmetric rocksalt phase (illustrated by dashed lines in Supplementary Figure 1b, c, d), we separate the unit cell into two inversion-partner sectors S_α and S_β (as indicated by pink and cyan in Supplementary Figure 1a) connected by the Te atom, and the local dipole fields are odd around the Te inversion center (Supplementary Figure 1d). However, the wavefunction is evenly distributed (guaranteed by inversion symmetry) among two inversion-partner sectors; together with the odd symmetry at the Te site. This leads according to Eq. (3) to a perfect compensation of the local dipole fields and thus vanishing Rashba effect in the centrosymmetric rocksalt phase.

However, in the non-centrosymmetric rhombohedral phase (illustrated by the solid lines in Supplementary Figure 1b, c, d) with Te being off-center, the corresponding wavefunction becomes segregated on one of these two sectors (S_β of Supplementary Figure 1e), while the local dipole fields around the displaced Te site is shifted along with the shifting of Te atom (Supplementary Figure 1d). These effects give the R-1 compound α-SnTe a residual dipole field felt by band states and thus give rise to a finite Rashba spin splitting according to the unified model described by Eq. (3), similar to that of R-2 spin splitting in $BaNiS_2$. This illustrates the identification of the two inversion-partner sectors in non-layered SnTe and shows how the R-1 spin-splitting originates from the local symmetries of the sectors rather than being a consequence of the global inversion symmetry breaking.

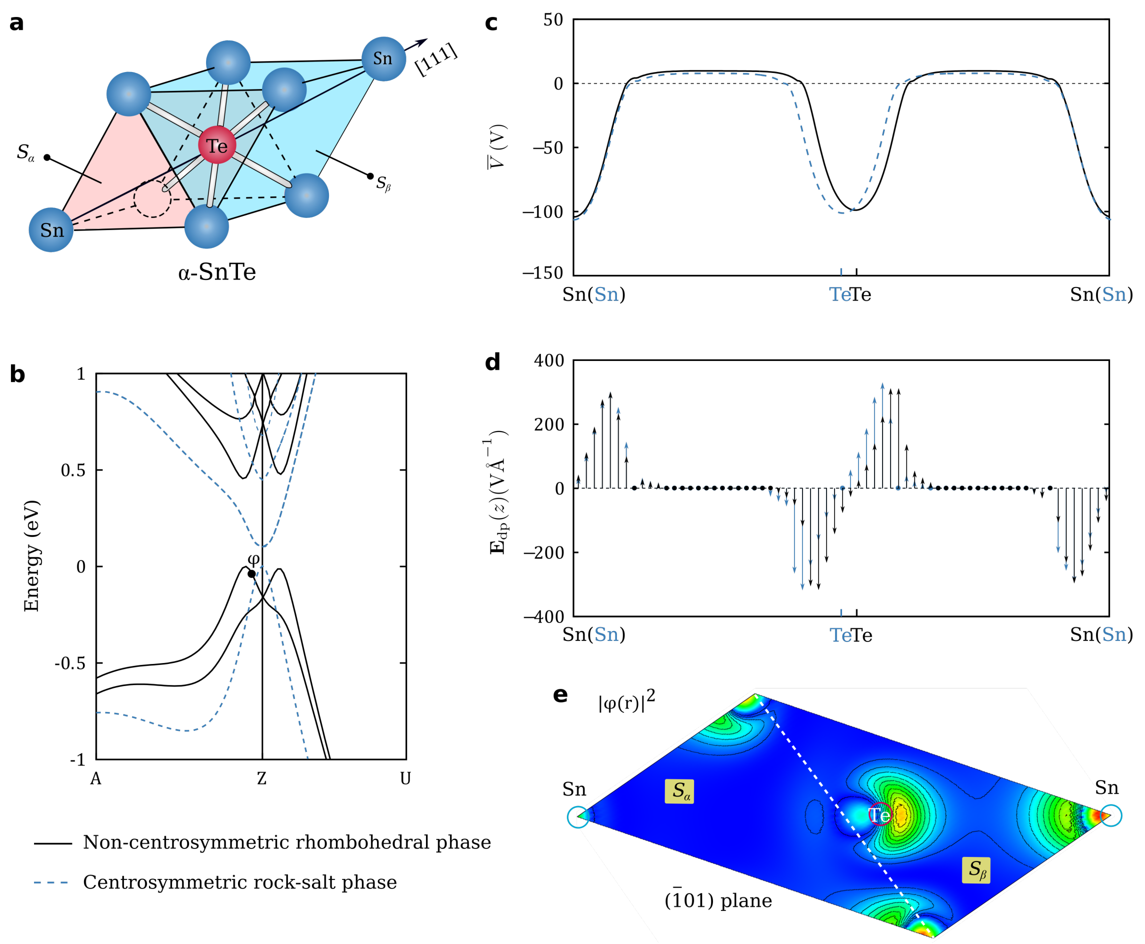

Supplementary Figure 1. Non-centrosymmetric rhombohedral α-SnTe having strong R-1 effect. a, Crystal structure of centrosymmetric α-SnTe identify with two inversion sectors S_α(pink domain) and S_β(cyan domain). The centrosymmetric phase transfers to a non-centrosymmetric rhombohedral phase as the Te atom displaced from Sn along [111] direction **b,** Band structure around the time-reversal invariant momentum Z along high symmetry A-Z and Z-U k-paths for both centrosymmetric (dashed lines) and non-centrosymmetric rhombohedral phase (solid lines). **c,** Averaged one-dimensional electrostatic potential (ionic + Hartree) along the [111] direction, and **d,** the corresponding electric dipole fields. **e,** Charge density in the $(10\bar{1})$ plane across the Sn and Te atoms for the state of the highest valence band at $\mathbf{k}_{Z-A}$ = (0.509, 0.5, 0.491) $(2\pi/a)$ with its corresponding state φ indicated in **b** by a black dot for the non-centrosymmetric rhombohedral phase.

Supplementary Note 2: Non-symmorphic screw axis symmetry enforces the four-fold degeneracy of the energy bands at $\bar{X}$ point of the tetragonal P4/nmm crystal

We show in Figure 2d that a four-fold degeneracy is present at $\bar{X}$ point of the tetragonal P4/nmm crystal. In non-magnetic material, such four-fold degenerate at time-reversal-invariant (TRI) **k** points can be enforced by a Hermitian symmetry operator that anti-commutes with the spatial inversion symmetry.[5] In the following, we will illustrate that, it is the non-symmorphic screw symmetry $\{C_{2x}|(a/2,0,0)\}$ -- which transforms (x,y,z) to $(x+\frac{a}{2},-y,-z)$ in position space -- that anti-commutes with the inversion symmetry and therefore ensures energy bands to be four-fold degenerate at TRI $\bar{X}$ points. Presence of such four-fold degeneracy is crucial for a non-vanishing R-2 spin splitting.

The coexistence of time-reversal symmetry operator T and the spatial inversion symmetry P in the nonmagnetic centrosymmetric crystals results in the double degeneracy (or spin degeneracy) of every energy bands. The symmetry operation PT transforms one spin component of each doubly degenerate bands to the other: $PT\psi(\mathbf{k},\boldsymbol{\sigma}) = \psi(\mathbf{k},-\boldsymbol{\sigma})$. Generally speaking, if L (including P) is a Hermitian symmetry operator of a centrosymmetric crystal, the transformation of the state ψ of the Hamiltonian under a symmetry operation L, which is $L\psi$, belongs to a state having the same eigenvalue as ψ. The Hermitian symmetry operator acting on the pair of degenerate states ψ and $PT\psi$ generates a new pair of degenerate states $L\psi$ and $PTL\psi$. If these two pairs of doubly degenerate states are nonequivalent, the energy bands will be four-fold degenerate. Under a different Hermitian symmetry operator $\mathcal{A}$ from L, these two pairs of doubly degenerate states $\{\psi, PT\psi\}$ and $\{L\psi, PTL\psi\}$ have different eigenenergies, i.e.,

$$\{A, A_{PT}\} \cap \{A_L, A_{PTL}\} = \emptyset \quad (1)$$

where A, A_{PT}, A_L, and A_{PTL} are the eigen-energies of ψ, ψ_{PT}, ψ_L, and ψ_{PTL} under the symmetry operator $\mathcal{A}$, respectively. At wavevectors k, which are invariant under both L and $\mathcal{A}$, the eigenenergies of these four states are degenerate, and thus the energy bands are four-fold degenerate at such k points.

At (and only at) TRI k points, we could take $L = P$, and the constrain on $\mathcal{A}$ in Supplementary Eq. (1) then becomes

$$\{A, A_{PT}\} \cap \{A_P, A_T\} = \emptyset. \quad (2)$$

Considering two-fold symmetries, which have two eigen-energies, are the most common Hermitian symmetry operators, we choose $\mathcal{A}$ as one of the two-fold symmetries and obtain $A = -A_P$. After inserting it into Supplementary Eq. (2), it is straightforward to have

$$\mathcal{A}P\psi = A_P P\psi = -PA\psi = -P\mathcal{A}\psi, \tag{3}$$

which reveals the anti-commutation between $\mathcal{A}$ and P,

$$\{\mathcal{A}, P\} = 0. \tag{4}$$

Therefore, a Hermitian symmetry operator $\mathcal{A}$, which anti-commutates with spatial inversion symmetry, ensures four-fold degeneracy of energy bands at TRI k points in the nonmagnetic centrosymmetric crystals. Such four-fold degeneracy is only available in non-symmorphic crystals because inversion symmetry P commutes rather than anti-commutes with all remaining symmetry operators in symmorphic crystals. The monolayer $BaNiS_2$ having the space group P4/nmm (No. 129) possesses a Hermitian operator of screw axis symmetry $\{C_{2x}|(a/2, 0, 0)\}$, under which the $\overline{\text{X}}$ point is invariant. It is easy to verify that $\{\mathcal{A}, P\} = 0$ taking $\mathcal{A} = \{C_{2x}|(a/2, 0, 0)\}$, and, therefore, the screw axis symmetry enforces the energy bands to be four-fold degenerate at TRI $\overline{\text{X}}$ point. Above, the non-symmorphic symmetry is essential for the anti-commutation relation and the four-fold degeneracy at certain TRI points (on Brillouin Zone boundary).

Supplementary Note 3: Restore the spin-split sector-segregated bands of monolayer $BaNiS_2$ from effective Hamiltonian

Based on the theory of invariants[6] we could derive the effective Hamiltonian and restore the DFT results of the spin-split sector-segregated bands. The derivation and the symmetry analyze incorporated will present us a more fundamental view of how non-symmorphic symmetry plays a role in determining the R-2 spin splitting as revealed in Figure 1. The monolayer $BaNiS_2$ has a non-symmorphic group P4/nmm (#129) consisting of 16 symmetry operators. Among these 16 operators, there are eight symmetry operators under which the $\overline{\text{X}}$-point is invariant (i.e., $\hat{R}\mathbf{k}_X = \mathbf{k}_X + \mathbf{G}$):

$$\{\boldsymbol{E}\,|\,\mathbf{0}\}: (\boldsymbol{x}, \boldsymbol{y}, \boldsymbol{z}) \rightarrow (\boldsymbol{x}, \boldsymbol{y}, \boldsymbol{z})$$

$$\{\boldsymbol{C_{2x}}\,|\,(\boldsymbol{a}/\mathbf{2}, \mathbf{0}, \mathbf{0})\}: (\boldsymbol{x}, \boldsymbol{y}, \boldsymbol{z}) \rightarrow (\boldsymbol{x} + \boldsymbol{a}/\mathbf{2}, -\boldsymbol{y}, -\boldsymbol{z})$$

$$\{\boldsymbol{C_{2y}}\,|\,(\mathbf{0}, \boldsymbol{a}/\mathbf{2}, \mathbf{0})\}: (\boldsymbol{x}, \boldsymbol{y}, \boldsymbol{z}) \rightarrow (-\boldsymbol{x}, \boldsymbol{y} + \boldsymbol{a}/\mathbf{2}, -\boldsymbol{z})$$

$$\{\boldsymbol{C_{2z}}\,|\,(\boldsymbol{a}/\mathbf{2}, \boldsymbol{a}/\mathbf{2}, \mathbf{0})\}: (\boldsymbol{x}, \boldsymbol{y}, \boldsymbol{z}) \rightarrow (-\boldsymbol{x} + \boldsymbol{a}/\mathbf{2}, -\boldsymbol{y} + \boldsymbol{a}/\mathbf{2}, \boldsymbol{z})$$

$$\{\boldsymbol{I}\,|\,\boldsymbol{0}\}: (\boldsymbol{x}, \boldsymbol{y}, \boldsymbol{z}) \rightarrow (-\boldsymbol{x}, -\boldsymbol{y}, -\boldsymbol{z}) \tag{5}$$

$$\{\boldsymbol{m_x}\,|\,(\boldsymbol{a}/\boldsymbol{2}, \boldsymbol{0}, \boldsymbol{0})\}: (\boldsymbol{x}, \boldsymbol{y}, \boldsymbol{z}) \rightarrow (-\boldsymbol{x} + \boldsymbol{a}/\boldsymbol{2}, \boldsymbol{y}, \boldsymbol{z})$$

$$\{\boldsymbol{m_y}\,|\,(\boldsymbol{0}, \boldsymbol{a}/\boldsymbol{2}, \boldsymbol{0})\}: (\boldsymbol{x}, \boldsymbol{y}, \boldsymbol{z}) \rightarrow (\boldsymbol{x}, -\boldsymbol{y} + \boldsymbol{a}/\boldsymbol{2}, \boldsymbol{z})$$

$$\{\boldsymbol{m_z}\,|\,(\boldsymbol{a}/\boldsymbol{2}, \boldsymbol{a}/\boldsymbol{2}, \boldsymbol{0})\}: (\boldsymbol{x}, \boldsymbol{y}, \boldsymbol{z}) \rightarrow (\boldsymbol{x} + \frac{\boldsymbol{a}}{\boldsymbol{2}}, \boldsymbol{y} + \frac{\boldsymbol{a}}{\boldsymbol{2}}, -\boldsymbol{z})$$

The rotation symmetry of these eight symmetry operators forms the little group D_{2h} of the $\overline{\mathrm{X}}$-point. The character table of D_{2h} is given in Supplementary Table 1. Note that $BaNiS_2$ as a non-magnetic crystal also contains the time reversal symmetry $\hat{T}$.

(1) The effective model Hamiltonian in the absence of SOC

The distinct band topology along $\overline{\mathrm{X}} - \overline{\mathrm{M}}$ and $\overline{\mathrm{X}} - \overline{\Gamma}$ of the monolayer $BaNiS_2$ without SOC shown in Figure 1c can be understood from the effective model Hamiltonian. Since the Rashba bands of our interesting around the $\overline{\mathrm{X}}$-point are mainly derived from the Ni *d*-orbits with $d_{x^2-y^2}$ and d_{z^2} character, the atomic *d* states of Ni atom $\phi_d(\boldsymbol{r})$, where *d* denotes a hybridization state of $d_{x^2-y^2}$ and d_{z^2}, are the reasonable choice as the basis of the invariant expansion. The Bloch basis $|\mathrm{Ni}_{\alpha(\beta)}, d, \mathbf{k}\rangle$ can be explicitly expressed as a Bloch sum of the local *d* states,

$$|\mathrm{Ni}_{\alpha(\beta)}, d, \mathbf{k}\rangle = \frac{1}{\sqrt{N}} \sum_{\mathrm{R}} e^{\mathrm{i}\mathbf{k}\cdot(\mathbf{R}+\boldsymbol{\tau}_{\alpha(\beta)})} \phi_d(\mathbf{r} - \mathbf{R} - \boldsymbol{\tau}_{\alpha(\beta)}) \tag{6}$$

Where $\mathbf{R}$ is the lattice vector and $\boldsymbol{\tau}_{\alpha(\beta)} = \pm(a/4, a/4, z_{\mathrm{Ni}_{\alpha(\beta)}})$ (a is the in-plane lattice parameter of the monolayer $BaNiS_2$) is the relative position of $\alpha(\beta)$ Ni atom within the primary cell for the origin setting in the middle of two Ni atoms.

The transformation property of the basis is ready to obtain upon application of the symmetry operations given in Supplementary Eq. (5). Taking the glide reflection operator $\{m_x|(a/2, 0, 0)\}$ as an example, we find that wavefunctions of the bands at the $\overline{\mathrm{X}}$-point segregated on Ni_α and Ni_β, respectively, have opposite parity under symmetry operation:

$$\{m_x|(a/2,0,0)\}|\mathrm{Ni}_{\alpha(\beta)}, d, \overline{\mathrm{X}}\rangle = \pm|\mathrm{Ni}_{\alpha(\beta)}, d, \overline{\mathrm{X}}\rangle \tag{7}$$

and thus, their interaction is strictly forbidden by symmetry. The energy equivalence but interaction forbidden of these two states causes an additional degeneracy besides the Kramers' degeneracy for bands at the TRI $\overline{\mathrm{X}}$-point, which is consistent with the observed four-fold degenerate bands at the $\overline{\mathrm{X}}$-point in the first-principles calculations. The same transformation rule could also apply to states along

$\overline{\mathrm{X}}-\overline{\mathrm{M}}$ direction, obtaining forbidden interaction between degenerate states segregated on two inversion-partner sectors and leading to the two-fold degeneracy of the energy bands in agreement with the first-principles calculation, as shown in Figure 1c.

The basis transforms, respectively, under the symmetry operations of $\{m_y|(0,a/2,0)\}$ and $\{I|0\}$ as

$$\{m_y|(0,a/2,0)\}|\mathrm{Ni}_{\alpha(\beta)},d,\overline{\mathrm{X}}\rangle = |\mathrm{Ni}_{\alpha(\beta)},d,\overline{\mathrm{X}}\rangle, \quad (8)$$

and

$$\{I|0\}|\mathrm{Ni}_{\alpha(\beta)},d,\overline{\mathrm{X}}\rangle = \pm \mathrm{i}|\mathrm{Ni}_{\beta(\alpha)},d,\overline{\mathrm{X}}\rangle. \quad (9)$$

Since the remaining symmetry operators of the D_{2h} are formed by the product of these three operators, the transformation properties of the basis under their operations are the combination of the results presented in Supplementary Eqs. (7)-(9). Without SOC, the time reversal symmetry $\hat{T}$ acts on the basis as a complex conjugate operator $\hat{K}$:

$$\hat{T}|\mathrm{Ni}_{\alpha(\beta)},d,\overline{\mathrm{X}}\rangle = \mp \mathrm{i}|\mathrm{Ni}_{\alpha(\beta)},d,\overline{\mathrm{X}}\rangle. \quad (10)$$

From Supplementary Equations (7)-(10) we learn that the representation matrixes of the symmetry operations $\{m_x|(a/2,0,0)\}$, $\{m_y|(0,a/2,0)\}$, $\{I|0\}$, and $\hat{T}$ acting on the basis $|\mathrm{Ni}_{\alpha(\beta)},d,\overline{\mathrm{X}}\rangle$ are τ_3,τ_0,τ_2, and $-i\tau_3\hat{K}$, respectively. Here, $\tau_0,\tau_1,\tau_2,\tau_3$ are 2×2 Pauli matrixes. The symmetrized matrix and irreducible tensors of symmetry operations of the D_{2h} point group up to second order in k are summarized in Supplementary Table 2 for the case without considering SOC.

According to the theory of invariant[6], the effective Hamiltonian must be invariant under all eight symmetry operations of the D_{2h} point group and, from Supplementary Table 2, we learn that all possible invariants up to second order in wavevector **k** at the $\overline{\mathrm{X}}$-point are $(C,k_x^2,k_y^2)\tau_0$ and $k_x\tau_1$. Neglecting the second order parabolic term, which will not contribute to spin splitting, the effective Hamiltonian of the spin splitting bands is therefore

$$\mathrm{H_{eff}} = tk_x\tau_1. \quad (11)$$

Here, t is a parameter that characterizes the strength of the interaction between two atomic d states located on two Ni atoms, respectively, within the primary cell. A diagonalization of Supplementary Eq. (11) yields the energy dispersion relation

$$E_\pm(\mathbf{k}) = \pm tk_x. \quad (12)$$

We, therefore, find that these two bands (without spin) arising from the atomic d orbitals of two Ni atoms, respectively, are degenerate when k_x is zero (i.e., along $\overline{\mathrm{X}}-\overline{\mathrm{M}}$ direction), but spits when k_x

is non-zero (i.e., along $\bar{\mathrm{X}}-\bar{\Gamma}$ direction). Such energy dispersion relation of the effective Hamiltonian explains well the results obtained from the first-principles calculations for the case without SOC, as shown in Figure 1c.

(2) The effective model Hamiltonian in the presence of SOC

Taking into account the spin degree of freedom, the basis must multiply the spin eigenstates $|\boldsymbol{\sigma}\rangle$ and becomes $|\mathrm{Ni}_{\alpha(\beta)}, d, \mathbf{k}, \boldsymbol{\sigma}\rangle$. Like in the case without SOC, the transformation properties of the basis (including spin) can be deduced by acting on them by symmetry operators The matrix representations of the symmetry operations of $\{m_x|(a/2,0,0)\}$, $\{m_y|(0,a/2,0)\}$, $\{I|0\}$, and $\hat{T}$ acting on the basis are $-i\sigma_1\tau_3, -i\sigma_2\tau_0, \sigma_0\tau_2$, and $\sigma_2\tau_3\hat{K}$, respectively. Here, $\sigma_0, \sigma_1, \sigma_2, \sigma_3$ are 2×2 Pauli spin matrices. Following the procedure of the theory of invariant, we obtain that all possible invariants $(C, k_x^2, k_y^2)\sigma_0\tau_0, k_x\sigma_0\tau_1, k_x\sigma_2\tau_3$, and $k_y\sigma_1\tau_3$ (given in Supplementary Table 3), under all eight symmetry operations of the D_{2h} point group for wavevector at the $\bar{\mathrm{X}}$-point. Neglecting the non-relevant second order parabolic term on spin splitting, the effective Hamiltonian of spin splitting bands around the $\bar{\mathrm{X}}$-point is written as

$$\mathrm{H}_{\mathrm{eff}} = tk_x\sigma_0\tau_1 + \alpha_{\mathrm{R}}^x k_x\sigma_2\tau_3 + \alpha_{\mathrm{R}}^y k_y\sigma_1\tau_3. \quad (13)$$

Here, α_{R}^x and α_{R}^y quantify the strength of the last two terms. In compared with the case without SOC, such effective Hamiltonian contains two additional terms induced by SOC, and hence known as Rashba terms. A diagonalization of Supplementary Eq. (13) yields the energy dispersion relation as

$$E_{\pm}(\mathbf{k}) = \pm\sqrt{\left(t^2 + {\alpha_{\mathrm{R}}^x}^2\right)k_x^2 + {\alpha_{\mathrm{R}}^y}^2 k_y^2}. \quad (14)$$

It is straightforward to learn that the four energy bands (including spin) along both $\bar{\mathrm{X}}-\bar{\mathrm{M}}$ and $\bar{\mathrm{X}}-\bar{\Gamma}$ directions spits into two doubly degenerate branches (branch A and branch B), which agrees with the results (shown in Figure 1d) obtained from the first-principles calculations for the case with SOC. It worth noting that since the off-diagonal elements are zero when $k_x = 0$ in the effective Hamiltonian given by Supplementary Eq. (13), the interaction between two atomic d states originating from two inversion-partner sectors are forbidden by the symmetry along $\bar{\mathrm{X}}-\bar{\mathrm{M}}$ direction ($k_x = 0$). The nonzero off-diagonal elements when $k_x = 0$ implies allowed mixture between two atomic d states originating from two inversion-partner sectors along $\bar{\mathrm{X}}-\bar{\Gamma}$. These results justify our findings of the wavefunction

segregation and fully delocalization on inversion-partner sectors along $\bar{X}-\bar{M}$ and $\bar{X}-\bar{\Gamma}$ directions, respectively.

Upon applying a small bias potential U perpendicular to the monolayer $BaNiS_2$, the potential dropping on two inversion-partner sectors lifts the degeneracy of both branch A and branch B, giving a justification for the observation of the first-principles calculation as shown in Figure 1e. The effect of the applied field can be described by $eU\sigma_0\tau_3$ added to Supplementary Eq. (13). Take it as a small perturbation, to first order in energy, immediately, the electric field induced splitting is $\sim 2\ eU$.

Supplementary Table 1. Character table of the D_{2h} point group.

D_{2h}	{E}	$\{C_{2z}\}$	$\{C_{2x}\}$	$\{C_{2y}\}$	{P}	$\{m_z\}$	$\{m_x\}$	$\{m_y\}$
A_g	1	1	1	1	1	1	1	1
B_{1g}	1	1	-1	-1	1	1	-1	-1
B_{2g}	1	-1	-1	1	1	-1	-1	1
B_{3g}	1	-1	1	-1	1	-1	1	-1
A_u	1	1	1	1	-1	-1	-1	-1
B_{1u}	1	1	-1	-1	-1	-1	1	1
B_{2u}	1	-1	-1	1	-1	1	1	-1
B_{3u}	1	-1	1	-1	-1	1	-1	1
$E_{1/2,g}$	2	0	0	0	2	0	0	0
$E_{1/2,u}$	2	0	0	0	-2	0	0	0

Supplementary Table 2. The symmetrized matrix and irreducible tensor of symmetry operations of the D_{2h} point group up to second order in k without considering SOC.

Irreps	Symmetrized Matrix	Irreducible tensor	$\{I\|0\}$	$\{m_x\|(a/2,0,0)\}$	$\{m_y\|(0,a/2,0)\}$	$\hat{T}$
A_g	τ_0	(C, k_x^2, k_y^2)	1	1	1	1
B_{3u}	τ_1	k_x	-1	-1	1	-1
B_{2g}	τ_2	-	1	-1	1	-1
B_{1u}	τ_3	-	-1	1	1	1

Supplementary Table 3 The symmetrized matrix and irreducible tensors of symmetry operations of the D_{2h} point group up to second order in *k* including spin.

Irreps	Symmetrized Matrix	Irreducible tensor	$\{I\|0\}$	$\{m_x\|(a/2,0,0)\}$	$\{m_y\|(0,a/2,0)\}$	$\hat{T}$
A_g	$\sigma_0\tau_0$	(C, k_x^2, k_y^2)	1	1	1	1
B_{3u}	$\sigma_0\tau_1$, $\sigma_2\tau_3$	k_x	-1	-1	1	-1
B_{2u}	$\sigma_1\tau_3$	k_y	-1	1	-1	-1
B_{1u}	$\sigma_0\tau_3$	U_z	-1	1	1	1

Supplementary References

1. Dimmock J, Melngailis I, Strauss A. Band structure and laser action in $Pb_x Sn_{1-x}$ Te. *Physical review letters* **16**, 1193 (1966).

2. Iizumi M, Hamaguchi Y, F. Komatsubara K, Kato Y. Phase transition in SnTe with low carrier concentration. *Journal of the Physical Society of Japan* **38**, 443-449 (1975).

3. Sugai S, Murase K, Kawamura H. Observation of soft TO-phonon in SnTe by Raman scattering. *Solid State Communications* **23**, 127-129 (1977).

4. Jantsch W. Dielectric properties and soft modes in semiconducting (Pb, Sn, Ge) Te. In: *Dynamical Properties of IV-VI Compounds* (ed^(eds). Springer (1983).

5. Liu Q, Zunger A. Predicted Realization of Cubic Dirac Fermion in Quasi-One-Dimensional Transition-Metal Monochalcogenides. *Physical Review X* **7**, 021019 (2017).

6. Bir GL, Pikus GE. Symmetry and strain-induced effects in semiconductors. (1974).
